# Trustworthiness of voting advice applications in Europe

**DOI:** 10.1007/s10676-024-09790-6

**Published:** 2024-08-12

**Authors:** Elisabeth Stockinger, Jonne Maas, Christofer Talvitie, Virginia Dignum

**Affiliations:** 1 Computational Social Science, ETH Zurich, Zurich, Switzerland; 2 Values, Technology and Innovation, Delft University of Technology, Delft, Netherlands; 3 School of Communication Research, University of Amsterdam, Amsterdam, Netherlands; 4 Computing Science, Umeå University, Umeå, Sweden

**Keywords:** Voting advice applications, AI ethics, Design for values, AI governance, Socio-technical systems, Responsible AI

## Abstract

Voting Advice Applications (VAAs) are interactive tools used to assist in one’s choice of a party or candidate to vote for in an upcoming election. They have the potential to increase citizens’ trust and participation in democratic structures. However, there is no established ground truth for one’s electoral choice, and VAA recommendations depend strongly on architectural and design choices. We assessed several representative European VAAs according to the Ethics Guidelines for Trustworthy AI provided by the European Commission using publicly available information. We found scores to be comparable across VAAs and low in most requirements, with differences reflecting the kind of developing institution. Across VAAs, we identify the need for improvement in (i) transparency regarding the subjectivity of recommendations, (ii) diversity of stakeholder participation, (iii) user-centric documentation of algorithm, and (iv) disclosure of the underlying values and assumptions.

**Supplementary Information:**

The online version contains supplementary material available at 10.1007/s10676-024-09790-6.

## Introduction

The debate surrounding regulation of artificial intelligence and its ethical implications is permeating the European technological landscape. Assessing conformity to ethics requirements, is, however, often challenging in nuances and complex contexts. Consider Voting Advice Applications (VAAs), interactive tools supporting the user in their electoral choice. VAAs are increasingly popular and influential in the European political landscape (Garzia & Marschall, 2014). They have the explicit goals of increasing political competence (Fossen & Anderson, 2014; Garzia et al., 2017; Munzert & Ramirez-Ruiz, 2021) and election turnout (Munzert & Ramirez-Ruiz, 2021; Mahéo, 2017; Munzert et al., 2021), and have been shown to impact vote choices (Munzert & Ramirez-Ruiz, 2021; Garzia & Marschall, 2012; Anderson & Fossen, 2014; Ramos et al., 2019).

Politics deals with questions that have no objective answer, or where the truth is not yet known (Barber, 2003). By extension, there is not yet a commonly agreed upon standard on how to evaluate its accuracy (Louwerse & Rosema, 2014; Padilla et al., 2021; Gemenis, 2013).

A growing literature basis elaborates on the methodological considerations underlying a VAA (Mendez, 2017; Guillermo Romero Moreno & Chueca, 2022; Walgrave et al., 2009; Louwerse & Rosema, 2014). Beyond the input given by the voter (Louwerse & Rosema, 2014; Padilla et al., 2021; Gemenis, 2013), architectural and design choices (e.g. how to phrase a particular statement in a questionnaire) significantly impact VAA output. Indeed, recommendations may differ by up to 90% depending on the distance function used to calculate the match between voter and party (Louwerse & Rosema, 2014). As argued by Fossen and van den Brink (2015), a VAA cannot “simply reflect what is at stake in the election by neutrally passing along information.” Rather, it structures information according to the developers’ interpretation of the electoral process and landscape. These limitations stress the importance of normative and ethical implications of VAAs. This area is still sparsely researched: Anderson and Fossen (2014) focus on the impact on the citizen’s conception of democracy, Fossen and Anderson (2014) discuss the normative implications of VAAs on political competence, political participation and democratic representation. The Lausanne Declaration on Voting Advice Applications (Anderson et al., 2014) presented a set of minimal standards for all VAAs upon which Padilla et al. (2021) based a set of normative criteria for VAAs with regards to increasing political competence. Where these normative criteria are aimed at competence, our study explores the question of whether VAAs meet required levels of trustworthiness as an algorithmic tool in the eyes of the users in a democratic context according to the Ethics Guidelines for Trustworthy AI (EGTAI). The EGTAI are developed by the High-Level Expert Group on Artificial Intelligence (HLEG) (High-Level Expert Group on Artificial Intelligence set up by the European Commission, 2019) at the request of the European Commission and are the basis of the recent AI Act (European Commission, 2021, p. 13). These guidelines serve as a general basis for what an algorithmic tool requires in order to be considered trustworthy by the standards of the European Union.

We contribute to the existing literature in three ways: Firstly, we conduct an ethics assessment of several VAAs used in European countries, representing different design strategies. To our knowledge, this article is the first to conduct a user-centric evaluation of VAAs, focusing on trustworthiness in the eyes of the electorate. To this end, we use a framework that is acknowledged by the democratic institutions of the countries hosting the VAAs and the respective elections, contributing to the democratic validity of a normative analysis of tools embedded in electoral processes. Secondly, we identify the abstract criteria that a trustworthy VAA must fulfill according to the EGTAI. Thirdly, we present a list of recommendations based on these issues to contribute to future VAA development efforts.

## Methods

We use a multi-case studies design to qualitatively evaluate the trustworthiness of VAAs within Europe. The cases are selected to represent the spectrum of common design variations. The Case selection Section provides a more detailed description.

There is a wealth of ethical and normative guidelines on AI, well summarized by Jobin et al. (2019), as well as a growing number of ethics standards and codes related to engineering practices such as IEEE 7000 (Systems and Software Engineering Standards Committee, 2021) and the IEEE P7000 series (e.g. Koene et al., 2018). As VAAs act in the context of democratic elections, they should be evaluated using methods that are acknowledged by the democratic institutions of the respective host countries. Since this addresses the trustworthiness of VAAs within Europe, we turn to the regulations and guidelines set out by the European Union (EU). There are several such regulations addressing digital technologies with the intent of strengthening an open, democratic and sustainable society, including the AI Act (European Commission, 2021) which contains some normative elements.

While there are some exceptions (Katakis et al., 2013; Guillermo Romero Moreno & Chueca, 2022), most VAAs do not employ machine learning methods. However, the AI Act uses the term ‘artificial intelligence system’ to refer to any software that can, for a given set of human-defined objectives, generate outputs such as predictions or recommendations, and that uses one or more of a set of techniques which include statistical approaches (European Commission, 2021, Article 3, point 1 and Annex I). VAAs fall under this broad definition of AI.

VAAs would likely be considered low-risk systems under the AI Act and be subject to light transparency rules at most. The Ethics Guidelines for Trustworthy AI (EGTAI) (High-Level Expert Group on Artificial Intelligence set up by the European Commission, 2019) relate to normative design, which is a representative and functional metric for trustworthiness especially in the absence of objective verifiability.

### Ethics guidelines for trustworthy AI (EGTAI)

Generally, AI systems should adhere to four fundamental principles: respect for human autonomy, fairness, explicability, and prevention of harm. This approach is akin to the well-established principles of bioethics (Beauchamp & Childress, 2001) with transparency in lieu of beneficence.

The EGTAI offer guidance on the implementation of trustworthy AI systems through seven key requirements:

1. Human agency and oversight,
2. Technical robustness and safety,
3. Privacy and data governance,
4. Transparency,
5. Diversity, non-discrimination and fairness,
6. Societal and environmental well-being, and
7. Accountability.

Each requirement is operationalized with an assessment list. A generic assessment list (High-Level Expert Group on Artificial Intelligence set up by the European Commission, 2020) was developed in collaboration with stakeholders across the private and public sector. This list should be used flexibly and adapted to a system’s particular context and sector. It should help organizations identify how a system can generate risks, and what measures may need to be taken to avoid or minimize those risks.

As there may be fundamental tensions between different principles and requirements, trade-offs should be continuously identified, evaluated, documented and communicated during the whole life cycle of the system. Especially situations involving vulnerable groups or asymmetries of power or information should be treated with due care and should include adequate measures to mitigate potential risks arising from the use of AI systems.

### EGTAI applied to VAAs

This section summarizes the EGTAI requirements and interprets them in the context of VAAs, forming an adapted, domain-specific assessment list. Each requirement is partitioned into a set of sub-requirements which collectively define the characteristics that a VAA should fulfill to be considered trustworthy.

#### R1: human agency and oversight

AI systems should support the user’s agency, foster fundamental rights, and be overseen by humans. Any possible impact on fundamental rights should be assessed prior to development of an AI system, and there should be mechanisms to receive external feedback. AI systems should respect and support the user in making more informed choices in accordance with their goals without subliminally influencing their behavior. Human oversight helps in ensuring that AI systems do not undermine human autonomy or cause other adverse effects.

VAAs target democratic elections, a context intricately tied to fundamental rights and highly sensitive to manipulation or distortion of information. They were inserted into a long-standing social context, and may have both intended and unintended consequences (Selbst et al., 2019). People and organizations may respond to this intervention in ways that are hard to predict without due and careful risk assessment. Users may not take recommendations into account consistently  (Stevenson, 2018; Christin, 2017) and show automation bias (Skitka et al., 2000; Citron, 2008; Selbst et al., 2019). VAAs may restrict the user’s conception of democracy and of the issues at hand (Fossen & Anderson, 2014). This tension may not be overcome, but potential negative effects should be acknowledged and critically examined. A trustworthy VAA should ensure human agency and oversight in the following ways:

R1.1: Prior to development, potential negative impacts on fundamental rights such as non-discrimination on the basis of political belief, freedom of expression and information, and privacy were assessed.
R1.2: Potential tensions and trade-offs between the different principles and rights were identified and documented.
R1.3: The potential influence on political opinions was evaluated in terms of its effect on the autonomy and informed decision-making of the user.
R1.4: Measures were taken to avoid overconfidence or overreliance on the VAA’s output on the users’ electoral decision and political processes.

#### R2: technical robustness and safety

AI systems should be resilient to attacks on all levels from data to physical and virtual infrastructure. There should be safeguards and fallback plans in case of problems to ensure that the system will minimize unintended consequences and errors. Results should be accurate, reproducible and reliable. Evaluating technical robustness and safety of VAAs requires an interpretation of the EGTAI from the perspective of declarative, rule-based models. Attacks specific to VAAs include the tampering of the algorithm to bias for or against a given party or candidate, the theft of user data for manipulation or defamation, or deceitful party or candidate answers.

As there is no consensus on how to measure the accuracy of VAAs, this requirement is particularly challenging. There are, however, several steps that can be taken to promote accurate, reproducible and reliable results. When VAAs rely on self-placement only, party representatives or candidates may be tempted to answer the questionnaire strategically rather than truthfully. Helsingin Sanomat, publishers of HS Vaalikone, observed a potentially strategic answering pattern by at least one party (Junkkari, 2023) with the aim of filling an empty corner on the VAA’s low-dimensional map. Similarly, the questionnaire may not be a representative mapping of the political space and the VAA may not perform comparatively across different political scenarios and for different user groups. Beyond these context-specific risks, VAAs are typically hosted as public-facing web services and vulnerable to cyber-attacks. The requirement of technical robustness and safety therefore comprises the following criteria:

R2.1: The placement of parties or candidates is conducted or verified independently.
R2.2: The questionnaire was validated with expert and user involvement.
R2.3: Measures were taken to prevent inaccuracies in user positioning.
R2.4: The security and resilience of the VAA against potential attacks has been thoroughly evaluated and addressed.
R2.5: There are no known issues in the main protocols, cipher suites, and standards that the websites support.
R2.6: There are no known issues with the libraries supported by the websites nor with cookie configuration.
R2.7: There is a clear procedure outlining how to handle errors in VAA functioning.
R2.8: The VAA was tested in different plausible political scenarios and for different user groups.

Vulnerability scanners by Qualys (Labs, 2024) and Pentest Tools (Tools, 2024) were used to evaluate requirements R2.5 and R2.6.

#### R3: privacy and data governance

Privacy is a fundamental right that necessitates a comprehensive data governance strategy. AI systems must guarantee privacy and data protection throughout their life cycle, including information provided by and inferred about the user.

VAAs handle highly sensitive data on a user’s political stances and therefore must protect the user’s identity. User data should not be collected for monetary gain. A trustworthy VAA therefore ensures the following:

R3.1: No sensitive data is collected that can be used to identify a user.
R3.2: The collection, analysis and provision of user data is not monetized.
R3.3: Privacy-preserving options are selected by default.

#### R4: transparency

The EGTAI require transparency of the data, the system, and the business models. The data sets and processes yielding the AI system’s output as well as the output itself should be documented to the best possible standards. This aids traceability and the identification of possible failure modes. The technical processes and output of a system should be understandable and traceable by humans. The design choices of the system, the rationale for deploying it, and the influence of an AI system on organizational decision-making processes should be transparent to the public. Humans have the right to be informed that they are interacting with an AI system. The AI system’s capabilities and limitations should be communicated to AI practitioners or end-users under consideration of the given use case.

Transparency to the user is highly relevant for any application providing input on democratic elections (Schumpeter, 1942; Habermas, 1989). Design decisions can shift the result drastically (Louwerse & Rosema, 2014; Padilla et al., 2021; Gemenis & Rosema, 2014). The issues that are important pre-election won’t necessarily stay relevant during the electoral period. Similarly, a candidate’s answers need not reflect the stances taken once elected (Schwarz et al., 2010).

To score highly in transparency, a VAA should therefore fulfill the following conditions:

R4.1: Several meaningful alternative result representations are provided.
R4.2: The user can see the answers per question and per party or candidate.
R4.3: Justification for candidate answers is available to the user.
R4.4: Design decisions regarding the questionnaire development including reasoning and actors involved are well-documented and accessible to the user.
R4.5: Design decisions regarding the algorithm including reasoning and actors involved are well-documented and accessible to the user.
R4.6: Possible benefits are clearly communicated to the user.
R4.7: Potential social limitations are clearly communicated, for example risk of confirmation bias by the user in interpreting the data.
R4.8: Limitations in discerning political candidate placements are clearly communicated to the user, for example candidates giving inconsistent or strategic answers or shifting their stances over time.
R4.9: Limitations regarding the subjectivity of design decisions are clearly stated.
R4.10: The exclusive focus on issue agreement regarding the topics covered by the VAA is clearly stated.
R4.11: Users are made aware of the algorithmic nature of the outcomes provided by the VAA.

#### R5: diversity, non-discrimination and fairness

The EGTAI stress the importance of inclusion and diversity throughout the AI system’s life cycle. The EGTAI point to unfair bias stemming from historically biased, incomplete or badly governed data sets as well as from the algorithm’s programming paradigm. The resulting system should be user-centric such that no user is disadvantaged due to age, gender, abilities or characteristics, stressing in particular accessibility for persons with disabilities. Stakeholders who may be directly or indirectly affected by a system should be consulted throughout the AI system’s life cycle.

**Fig. 1:**
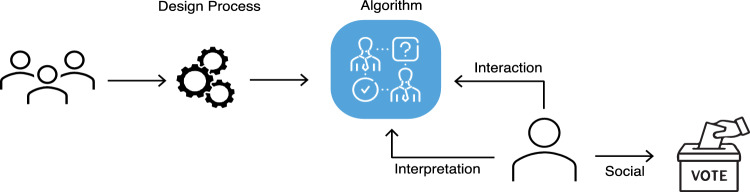
Possible sources of bias in voting advice applications

VAAs are susceptible to bias in several phases including the design process, the user’s interaction with the VAA’s interface (typically the questionnaire), the user’s interpretation of the recommendation, and the user’s subsequent vote in an upcoming election (Fig. 1). Besides non-discrimination of users, VAAs must also ensure that no candidates or parties are disadvantaged. A trustworthy VAA therefore fulfills the following sub-requirements:

R5.1: A VAA clearly communicates whether all candidates are included in the results and, if necessary, which ones are excluded.
R5.2: The questionnaire design process includes stakeholder voices representing users, experts, and parties of candidates.
R5.3: Stakeholders involved in the design process have decisive power.
R5.4: Stakeholder involvement in development considers diversity across different socio-demographic or political groups.
R5.5: Steps are taken to accommodate all users, and a possible failure to do so is acknowledged, along with a possible resulting disadvantage in electoral representation.
R5.6: Potential biases during development, deployment, and use are tested and monitored.
R5.7: There is a clear mechanism to allow flagging of issues related to bias, discrimination or poor performance.
R5.8: Fairness of the VAA’s output has been defined and adequately quantified.
R5.9: The VAA accommodates a wide range of individual preferences and abilities.
R5.10: The VAA is usable by those with special needs or disabilities as well as by those at risk of exclusion.
R5.11: The VAA is available in several languages beyond the national one.

#### R6: societal and environmental well-being

The EGTAI discuss potentially negative effects regarding environmental well-being, work and skills, as well as society at large and democracy. The effects of an AI system on social agency, relationships or social skills must be carefully monitored. On a societal perspective, due consideration should be given to situations relating to the democratic process including political and electoral decision-making.

VAAs modulate information in the political domain and can be a factor in determining the leaders who shape the system. Discrepancies in access to the system may disadvantage certain user groups over others. Similarly, the focus on issues where one party’s positioning is more popular in the VAA’s user base may lead to its disproportional benefit.

Direct environmental and work-related impact of VAAs is insignificant, though VAAs may create impact through their effect on electoral behavior. A possible secondary effect of the popularity of VAAs is the strategic self-placement of parties both in answering the questionnaire and in defining their stances, an encouragement of issue voting and promissory voting (Ladner, 2016). VAAs may also collaborate with researchers to create benefits for human knowledge.

This requirement includes the following conditions:

R6.1 The VAA topically covers issues that may result in harm to essential areas such as environment, societal welfare, rule of law, and democracy.
R6.2 The societal impact of using the VAA was assessed, including possible indirect effects on stakeholders and society at large.
R6.3 Steps were taken to minimize potential societal harm.
R6.4 The impact of VAA use on election outcomes across electoral contexts is documented and communicated to the user.
R6.5 Potential long-term effects on political representation, value allocation, or on the importance of the several factors used to take a political decision are communicated transparently.
R6.6 The VAA contributes to academic research.

#### R7: accountability

The EGTAI require that mechanisms be put in place to ensure responsibility and accountability for the AI system and its outcome during development, deployment and use. The algorithms, data and design processes must be auditable by internal and external parties and the resulting evaluation reports should be available. Negative impacts should be identified, assessed, documented, and minimized. Tensions between the listed requirements may necessitate trade-offs, which should be addressed rationally and methodically. Risks to ethical principles should be explicitly acknowledged, evaluated, and documented. Accessible mechanisms should be foreseen to redress a possible adverse impact with particular attention to vulnerable persons and groups.

VAAs must ensure a responsible treatment of democratic electoral processes and follow the recommendations described by the EGTAI carefully. An ideal VAA, therefore, fulfills the following criteria:

R7.1: Relevant interests and values implicated by the AI system and potential trade-offs between them are acknowledged and evaluated.
R7.2: Users, developers or candidates can report potential vulnerabilities, risks or biases in the AI system in a clearly defined and easily accessible manner.
R7.3: There is a clear procedure to deal with possible failures or security breaches of the VAA.
R7.4: If candidates can redress their placement on issues, there must be a clearly defined procedure.
R7.5: Ethical concerns and accountability measures are overseen with external guidance or by third-party auditing processes throughout the system’s life cycle.
R7.6: The VAA conducted risk training including potentially applicable legal frameworks.
R7.7: There is an ethics board or comparable institution responsible for reviewing potential unintended societal effects.

### Evaluation, quantification and scoring

The EGTAI do not necessarily imply that all information about business models and intellectual property rights related to AI systems should be openly available. However, as VAAs directly relate to democratic elections, their design, output, and raison d’être must be transparent to the electorate to allow trust (Héritier, 2003; Schumpeter, 1942; Habermas, 1989). We therefore restrict our document analysis exclusively to secondary and unsolicited data that is publicly available and easily retrievable. In particular, we consider information hosted on the respective web applications and published by the developing institutions. Table A1 provides more details on the corpus under analysis.

Each of the seven key requirements $R \in \{ R1, R2, ...R7 \}$ outlined above is comprised of a set of sub-requirements. Each sub-requirement $r \in R$ is measured on a discrete scale from 0 to *n*, where *n* depends on the nature of the question (see Table A4) and is normalized to 1 in the calculation of compliance scores.

The compliance score for a requirement *R* is the ratio of achieved normalized points of total achievable points. Formally, compliance *c* with a requirement *R* is defined as:

1 $$c(R, v) = \frac{1}{|R|}\sum _{r \in R} \frac{p(r,v)}{max(r)}$$

where *max*(*r*) returns the maximum number of points reachable for sub-requirement *r*, and *p*(*r*, *v*) returns the points achieved by the VAA *v*.

## Case selection

VAAs can differ both in their design and in the broader context they are submerged in. In terms of context, VAAs may be institutionalized features of the political landscape. For example, the German Wahl-O-Mat is operated by a federal government agency. With 15.6 million usages before the 2017 elections, it is the most popular VAA in absolute numbers (Garzia et al., 2017). Some VAAs are developed by politically neutral non-profit organizations which may be financed by parties or candidates (e.g. the Swiss Smartvote) or subsidized by governmental bodies (e.g. the Dutch StemWijzer). Other VAAs are developed by private corporations such as the Dutch research agency Kieskompas or the Danish newspaper Altinget. Especially in Finland and Sweden, many prominent media outlets operate their own VAAs at comparable levels of popularity: at least 13 VAAs were available for the Finnish parliamentary elections in 2023 (hyviaasioita.fi, 2023), most hosted by large media houses.

**Fig. 2:**
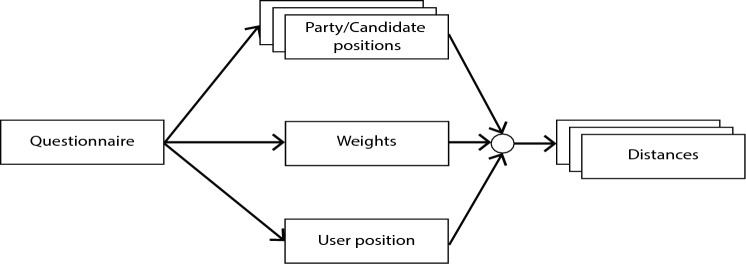
A generic model of a VAA matcher algorithm

In terms of design, most VAAs follow a common operating principle (Fig. 2) (Garzia & Marschall, 2019): A questionnaire is used to find the user’s and the candidate’s positions on policy statements. In some cases, the user can additionally specify relative weights. Subsequently, the distance in between the user and each candidate is calculated using the weight matrix. The resulting distances map to the agreement score of each party or candidate and are presented to the user either as a ranking or graphically.

There are several design variations beyond this common model. For example, the social VAA “Choose4Greece” provides community-based advice based on the voting intention of similar users (Katakis et al., 2013). Romero Moreno, Padila and Chueca (2022) present a method which learns saliency weights and issue-voting space from user data, and Bachmann et al. (2024) introduce an adaptive questionnaire approach that selects subsequent questions based on users’ previous answers. However, these methods are rare in popular VAAs, which mostly vary along the characteristics such as the size of the questionnaire, possible answer categories or question weights. Table A2 provides more details on common variations.

VAAs are commonly sorted into one of three families according to varying complexity, party placement, and result presentation. Our case selection includes at least two instances per family, reflecting this diversity in design and context (Table 1). Each of these families is based on a pioneering VAA, which is contained in our case selection  (Stefan & Diego, 2014):

- *StemWijzer*: characterized by simplicity and user-friendliness, usually offering three answering categories. Parties place themselves on the questionnaire items, results are presented as a ranked list. This family includes the German Wahl-O-Mat.
- *Kieskompas*: parties are placed by experts, often using document analysis. Results are mostly presented as a 2D map. The Swedish Aftonbladets valkompass leans towards this family.
- *Smartvote*: uses comparatively complex algorithmic methods and offers several alternative result visualizations. The Finnish HS Vaalikone is based on this model.

We also included the Swedish SVT Nyheters valkompass which displays relatively high algorithmic complexity but presents results only as a ranked list. Table A1 provides further details on the institutions developing these VAAs on the respective elections. Their design characteristics are summarized in more detail in Table A3.

**Table 1:** Selected VAAs and their distinguishing features

	Family leaning	Type of developing institution	Complexity	Political placement by	Result presentation
StemWijzer	StemWijzer	Non-profit	Low	Parties	Ranking
Kieskompas What2Vote	Kieskompas	Private research agency	Medium	Experts^a^	Spatial map
Smartvote	Smartvote	Non-profit	High	Candidates	Ranking, spatial map, radar plot
Wahl-O-Mat	StemWijzer	Federal agency	Low	Parties	Ranking
Aftonbladets valkompass	Kieskompas	Media company	Low	Hybrid	Ranking, spatial map
HS Vaalikone	Smartvote	Media company	High	Candidates	Ranking, spatial map, additional value axes
SVT Nyheters valkompass	Smartvote/StemWijzer	Media company	Medium	Parties	Ranking

^a^Placement may also involve party representatives, see Kieskompas What2Vote for details

**Fig. 3:**
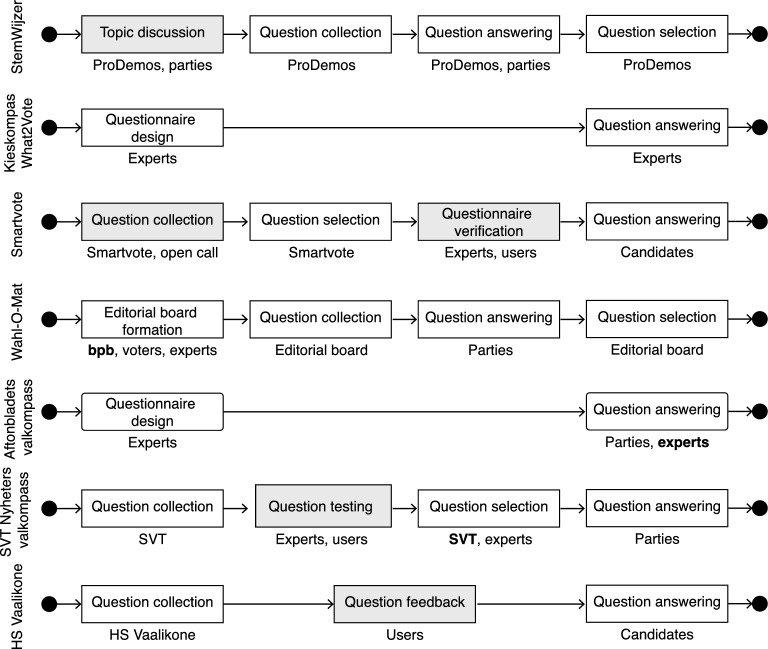
The stages of the design process of the questionnaire as documented by the VAAs. Non-decisive steps are shaded in grey. The stakeholders involved in each step are printed below each stage. If several stakeholders are involved in a design stage and one has decision power, they are listed in bold

While this selection is based on the design outcome, EGTAI scores may also reflect the design process. This process and the actors involved therein are summarized in Fig. 3.

### StemWijzer

StemWijzer provides information on the party-level. At the start of each questionnaire iteration, StemWijzer editors consult party election programs and discuss the most important topics in a workshop with the political parties and within a voter panel  (StemWijzer, 2023; Bamberg et al., 2021). The developers use this input to generate a list of about sixty statements. All parties participating in the given election can indicate whether they agree or disagree with the statement, or whether they take a neutral position. Thirty statements from this list to which parties have taken clearly different stances are included in the questionnaire (StemWijzer, 2023).

### Kieskompas What2Vote

The Kieskompas methodology has been extensively described by Krouwel and van Elfrinkhof (2014) (the first author is the founder of Kieskompas):

1. selection of parties or candidates to be included,
2. selection of authoritative sources to use for party placement,
3. identification of salient issues to include using computer assisted text analysis with expert opinion,
4. framing of propositions about these issues,
5. party coding based on the selected sources using computer-assisted text analysis and expert opinion,
6. party authorization of the coding decisions (final decision is retained by the coding team),
7. definition of rules to governing voter and party position comparisons, and
8. development of the multidimensional map of the political space.

However, it is unclear whether this description remains valid for the 2023 Kieskompas What2Vote. The VAA website describes the process of political positioning merely as an independent deduction from programs and statements of party leaders based on academic methods where parties have no say (Kieskompas BV, 2024a).

### Smartvote

Smartvote provides information for each candidate. Results are also available on the level of candidate lists. Questions are solicited in an open call. Input may stem from parties, interest groups, citizens and media on top of the editors themselves. The questions are selected by the editors under consideration of divisiveness, topicality and general interest. Experts from academia and users provide feedback. All candidates participating in the election may then provide their answers (Politools, 2023). Beyond political issues, some questions are designed to elicit the users’ values. For example, in the questionnaire for the National Council elections in Switzerland 2019, Smartvote asked about the user’s position on the statement: “Someone who is not guilty has nothing to fear from state security measures” (Politools, 2023). Smartvote provides three separate result presentations: a scored ranking, a 2-dimensional map, and a 5-dimensional radar plot, each using a different calculation method. The candidate closest to a user may differ in between result presentation.

Smartvote allows users to create profiles (Politools, 2023). Answers to reused questions will be saved and auto-filled in subsequent questionnaires. This may be due to the elements of direct democracy in Switzerland, calling voters to the urns more frequently than in other countries and adding incentive to convenience over privacy.

### Wahl-O-Mat

Wahl-O-Mat is a popular VAA of the StemWijzer family making heavy use of participatory design. The questionnaire design process is started by forming an editorial board including representatives from the Federal Agency for Civic Education (Bundeszentrale für politische Bildung, bpb), young voters and experts. The editorial board jointly develops a list of 80 potential questionnaire items during a 3-day workshop using party and electoral programs as well as candidate statements. After party representatives answer these items, the editorial board selects 38 items for the Wahl-O-Mat in a second workshop such that important and divisive topics are covered. Party representatives are not involved in questionnaire development.

### Aftonbladets valkompass

Aftonbladets valkompass is developed in collaboration with the Society, Opinion and Media (SOM) Institute of the University of Gothenburg and with Altinget (Altinget, 2023), a politically neutral online newspaper with extensive experience in VAA development and research.

Aftonbladets valkompass follows the Kieskompas method of party placement described by Krouwel and van Elfrinkhof (2014): The SOM institute carries out an independent coding of party positions in parallel to parties placing themselves in relation to the questionnaire items. The placement results are then compared and discussed between the experts and party representatives. In case of disagreement, the SOM institute decides the final ranking. (Ekman & Aftonbladet, 2022). Altinget contributes with the calculation of results and methodical advice.

### HS Vaalikone

Some of the VAA development process is described in accompanying newspaper articles, other sources are mostly academic (Isotalo, 2021).

HS Vaalikone’s design was significantly altered for the 2023 elections, now focusing on value questions in addition to questions targeting concrete political issues. This decision is based on the drastic change of importance of pre-election issues during election cycle of 2019–2023, when the COVID-19 pandemic and land war in Europe dominated the political discussion  (Nurmela & Hulst, 2023). For example, for the parliamentary elections in Finland on Sunday 2 April 2023, HS Vaalikone asked for the user’s stance on the issue: “Finland becoming more multicultural and diverse than before is a good thing” (Helsingin Sanomat, 2023).

Issue questions are compared in high-dimensional space (such that each issue question is considered a separate dimension) with Manhattan distance. The representation space of value questions is reduced to a set of ideological dimensions using Confirmatory Factor Analysis (Brown, 2015) which are compared using Euclidean distance (Nurmela & Hulst, 2023).

With its algorithmic complexity of value mapping and the different result representations, HS Vaalikone leans towards the Smartvote family of VAAs. Distance is calculated on a candidate level (Nurmela & Hulst, 2023), though results can be aggregated to the level of parties. All candidates participating in an election are invited to fill the questionnaire and be represented in the VAA.

### SVT Nyheters valkompass

Both the SOM institute and Altinget are involved in the SVT Nyheters valkompass design process as well, though as opposed to the case of Aftonbladets valkompass they remain in advisory roles.

In a first step, SVT Nyheters valkompass collects around 100 questionnaire items based on party policies and topical issues. These items are condensed down to 50 using panel tests conducted by SOM institute as well as user tests. Other advisors involved in this stage include external political scientists and experts from Altinget. It is not entirely clear from our analysis at what stage party representatives answer these questions, though it seems to be after finalization of the questionnaire.

There are three different types of items in SVT Nyheters valkompass. *Propositions* use a four-level Likert scale to indicate agreement, a *priority question* allows to select up to three general areas to be prioritized (such as “Law and Order” or “Elderly care”), and single-choice *range answers* (such as “How much of the forest in Sweden should be protected”) with five options ranging from *much more* to *much less*. The match is calculated by the percentage of the maximum agreement score aggregated over all items (Andersson, 2019).

## Results

**Fig. 4:**
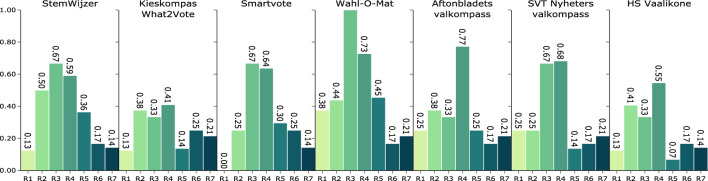
Aggregated compliance of each VAA with the EGTAI key requirements calculated according to Eq. 1. The bars are colored according to the requirement they represent

The points achieved per VAA and sub-requirement are given in detail in Table A4. Numeric results are provided in Table A5. The aggregated compliance scores of each VAA per requirement are shown in Fig. 4.

### R1: human agency and oversight

No VAA scores highly on requirement R1 under the adapted EGTAI assessment list, providing neither information on a human rights impact assessment (R1.1) nor on an evaluation of the potential influence on autonomy or informed decision-making of the user (R1.3).

Wahl-O-Mat is faced with a unique challenge regarding trade-offs between different principles and rights: in 2020 the German Federal Office for the Protection of the Constitution classified the faction “Der Flügel” within the party “Alternative für Deutschland” (AfD) as a “right-wing extremist endeavor against the free democratic basic order” and not compatible with the Basic Law (Tagesschau, 2020; Bundesministerium des Inneren und für Heimat, 2021). The question of whether to provide voting advice for this party involves the principles of prevention of harm, fairness, and human autonomy. The Wahl-O-Mat alludes to this tension in their FAQ, indicating that recommended parties may be considered extremist and linking to the respective reports (Bundeszentrale für politische Bildung, Landeszentrale für politische Bildung Bremen, 2023). HS Vaalikone mentions possible negative effects of transparency on security in a newspaper article (Salminen & Nurmela, 2023) and in an academic paper on the initial VAA development (Isotalo, 2021) (R1.2).

Aftonbladets valkompass and SVT Nyheters valkompass aim to prevent over-reliance on the VAA result by referring to factors that may influence an electoral decision other than issue agreement (Nyheter, 2022; Ekman & Aftonbladet, 2022) (R1.4). Wahl-O-Mat extensively discusses limitations of the VAA result, referring to possible unfaithful answers and differences in interpretations of questions. Smartvote, rather than attempting to prevent over-reliance on the VAA, describe their recommendation as a purely mathematical and politically neutral measure  (smartvote, 2019) StemWijzer refers to itself as a tool to discover substantive differences between parties and encourages the voters to think for themselves. Kieskompas What2Vote goes a step further by not providing a voting proposal at all. Rather, results are displayed only as projections on a 2-dimensional map such that users need to consider the range of parties close to them alongside ideological dimensions (R1.4). HS Vaalikone does not mention the topic of over-reliance.

### R2: technical robustness and safety

The VAAs follow different strategies when it comes to the verification of political placement (R2.1). VAAs of the StemWijzer and Smartvote families rely on self-placement by candidates or party representatives. A podcast episode on StemWijzer states that answers given by party representatives are verified by the editors (Bamberg et al., 2021). The public code repository of HS Vaalikone contains some validation of two of the ideological dimensions (Left to Right and Green/Alternative/Libertarian to Traditional/Authoritarian/Nationalist) and aggregated party placement through expectation matching, though lacking in detail and rigor (Nurmela & Hulst, 2023). User-facing information on the Kieskompas What2Vote method of party placement is sparse, referring only to document analysis without party involvement (Kieskompas BV, 2024a). Aftonbladets valkompass goes into more detail: Academic experts of the SOM institute carry out an independent coding of party positions while parties are asked to place themselves in parallel with reference to official party materials. Divergences between party and expert answers are discussed bilaterally. In case of disagreement, the SOM institute decides the final ranking (Ekman & Aftonbladet, 2022).

Smartvote and StemWijzer use expert and user feedback in the validation of the questionnaire  (Politools, 2023; Bamberg et al., 2021) (R2.2). HS Vaalikone requests user feedback through news articles (e.g. Salminen (2022); Nalbantoglu and Salminen (2022)). SVT Nyheters valkompass points to several non-binding validation tests for the questionnaire and a collaboration with external advisors and experts. Kieskompas What2Vote refers to a collaboration with the National and Kapodistrian University of Athens to monitor questionnaire development (Kieskompas BV, 2024a). Aftonbladets valkompass does not go into detail on questionnaire development beyond naming the responsible actors (Ekman & Aftonbladet, 2022). Similarly, Wahl-O-Mat mentions no validation beyond the presence of experts and users on the editorial board (Bundeszentrale für politische Bildung, Landeszentrale für politische Bildung Bremen, 2023).

Wahl-O-Mat and StemWijzer don’t give recommendations to users who go through the questionnaire too fast (R2.3).

No VAA elaborates on questions of security or resilience against attacks (R2.4). Most VAAs show some security issues as shown in Table A6 (R2.5, R2.6). Smartvote in particular supports several technologies with known vulnerabilities, which is particularly concerning in light of their storage of questionnaire answers. The StemWijzer server is configured securely, though at time of analysis the website used a library with a known vulnerability to cross-site scripting, and Aftonbladets valkompass and Kieskompas What2Vote display insecure cookie configurations.

No VAA provides a fixed policy of dealing with errors such as the faulty placement of candidates or duplicated questions (R2.7), though HS Vaalikone provides anecdotal information through a newspaper article (Salminen, 2023). Similarly, no VAA provides information on tests addressing different political scenarios or user groups (R2.8).

### R3: privacy and data governance

Generally, privacy and data governance policies of VAAs may range in focus from user privacy towards data analysis.

StemWijzer collects only technical data and aggregate usage data (R3.1). Data is stored only in aggregate form to prevent the inference on the individual user (R3.3).

Wahl-O-Mat minimize the data they collect, storing only technical logs (Bundeszentrale für politische Bildung, 2023) and achieving full points on this requirement (R3.1–R3.3).

Smartvote collects only technical data necessarily. Personal data relating to the user profile and participation data are stored separately (R3.1, R3.3). User and aggregated usage data is stored separately and within the EU (R3.3). Smartvote generates income by offering services mainly to media partners and other organizations including data analysis and surveys  (Politools, 2023) (R3.2).

Kieskompas is open regarding privacy and data governance, providing a separate, user-friendly FAQ on issues relating to privacy (Kieskompas BV, 2024c). Automatically collected data include quantitative usage data and information regarding the device used to access the site (R3.1). Users may share their email address, which is stored separately from questionnaire answers and demographic questions such that filling in the VAA remains anonymous (R3.3). As a private research agency, Kieskompas has a direct economic interest in the VAA data (R3.2).

SVT Nyheters valkompass stores quantitative usage data. The policy on aggregated user data is unclear - the FAQ mainly states that SVT does not save any personal data linked to how individual visitors answered the various questions (R3.1).

Neither Aftonbladets valkompass nor HS Vaalikone store questionnaire answers, but will, if permitted by the user, collect general user data for the purposes of targeted advertisement (R3.2).

### R4: transparency

The result presentations differ across VAA families (R4.1). StemWijzer, Wahl-O-Mat and SVT Nyheters valkompass provide a scored ranking of candidates while Kieskompas What2Vote offers only a 2-dimensional map. The other VAAs display both alternatives. StemWijzer additionally provides a 5-dimensional radar plot, and HS Vaalikone offers a set of 1-dimensional value axes in addition.

All VAAs in this study provide the answers per question and candidate (R4.2), though Smartvote does not support the comparison of party positions per question. VAAs using self-placement allow candidates to justify their answers to the users (R4.3). Aftonbladets valkompass and Kieskompas What2Vote refer to the public documents used in party placement.

Documentation on questionnaire design varies strongly across VAAs. Smartvote, SVT Nyheters valkompass and StemWijzer provide methodological details (R4.4). Although the method used by Kieskompas is described extensively in scientific literature (Krouwel & van Elfrinkhof, 2014), this information is not easily accessible to the layperson. Similarly, Aftonbladets valkompass lists the responsible actors but does not describe the method of questionnaire development itself, and Wahl-O-Mat provides little information on the content and form of workshops of the editorial board. HS Vaalikone does not go into detail on the question selection process.

The VAAs also differ in how they assure algorithmic transparency (R4.5). Wahl-O-Mat, StemWijzer and Aftonbladets valkompass rely on relatively simple algorithms which they describe accessibly to the user (Bundeszentrale für politische Bildung, Landeszentrale für politische Bildung Bremen, 2023; StemWijzer, 2023; Aftonbladet, 2016). Smartvote uses different calculation methods with varying levels of complexity for its result representations: Candidates are projected onto the 2-dimensional map using principal component analysis, where axes are labeled post-hoc (smartvote, 2015a). The projection onto the radial plot, on the other hand, is calculated by assigning the questions within the questionnaire to predefined axes in an n-to-n relationship (smartvote, 2015b). The methodology for each representation and the meaning of the projections are well-described (smartvote, 2019, 2015b, a).

HS Vaalikone and SVT Nyheters valkompass provide their matching algorithms on public repositories (Andersson, 2019; Nurmela & Hulst, 2023). Many design decisions are only accessible to those able to read the open-source code, for example the respective weighting in between and the result aggregation of propositions, range questions and priority questions in the case of SVT Nyheters valkompass as well as result aggregation, dimensionality reduction and different treatment of issue and value questions in HS Vaalikone. Some design choices remain hard to interpret even for those able to read the code. Kieskompas What2Vote does not provide details to the user.

All VAAs openly communicate the algorithmic nature of advice (R4.11). Smartvote, SVT Nyheters valkompass and Aftonbladets valkompass explicitly list the benefits provided by the VAA (R4.6).

Limitations are discussed only in passing. Social limitations such as confirmation bias (R4.7) are mentioned by the StemWijzer developers in a podcast episode (Bamberg et al., 2021) and implied by the choice of Kieskompas What2Vote not to provide an explicit recommendation. Limitations in candidate placement (R4.8) are mentioned by HS Vaalikone in their discussion of the rapid change of political priorities within the election cycle  (Nurmela & Hulst, 2023). Wahl-O-Mat clearly touches on the possibility that candidate answers may not be representative (Bundeszentrale für politische Bildung, 2023). The subjectivity of VAA recommendations (R4.9) is mentioned by Aftonbladets valkompass in their elaboration on not allowing the user to specify weights due to the danger or arbitrary weighting (Ekman & Aftonbladet, 2022). Wahl-O-Mat, SVT Nyheters valkompass, StemWijzer and HS Vaalikone reject objectivity of results, advising the user to seek out additional sources of information. Smartvote and Kieskompas What2Vote, on the other hand, claim to provide unbiased results (smartvote, 2019; Kieskompas BV, 2024a). SVT Nyheters valkompass and Aftonbladets valkompass elaborate on the limitation of voting only based on issue agreement (R4.10), listing several other favors that could play into an electoral choice (Nyheter, 2022; Ekman & Aftonbladet, 2022). Wahl-O-Mat discusses the difference in issue saliency across different people (Bundeszentrale für politische Bildung, Landeszentrale für politische Bildung Bremen, 2023), while Smartvote and HS Vaalikone attempt to map value alignment in addition to issue agreement.

### R5: diversity, non-discrimination and fairness

While all parties running in the given election are invited to participate in StemWijzer and HS Vaalikone, the results do not explicitly reflect which parties have declined to do so and are therefore not listed (R5.1). Smartvote displays these candidates on the results page as missing. All parties are represented in Wahl-O-Mat. SVT Nyheters valkompass, Aftonbladets valkompass and Kieskompas What2Vote communicate their inclusion criteria on their FAQ.

The VAAs differ strongly in how stakeholders are involved in the questionnaire design process (R5.2, R5.6), though none refer to stakeholder diversity (R5.4) or consider that some parts of the population may not be accommodated (R5.5). Smartvote elicits questions in an open call. As in the case of SVT Nyheters valkompass, users and experts are involved subsequently (Politools, 2023; Nyheter, 2022). Wahl-O-Mat includes young voters and experts within the editorial board (Bundeszentrale für politische Bildung, Landeszentrale für politische Bildung Bremen, 2023). StemWijzer involves parties in topic selection and provides an email address for users to suggest questions. HS Vaalikone asks for user feedback in newspaper articles (Salminen, 2022; Nalbantoglu & Salminen, 2022). The Aftonbladets valkompass questionnaire is developed entirely by academic experts, and Kieskompas What2Vote works with a team of the National and Kapodistrian University of Athens (Kieskompas BV, 2024a).

Wahl-O-Mat shares the decision power amongst its editorial board (R5.3), Aftonbladets valkompass separates responsibilities by relying entirely on the SOM institute for questionnaire development. All other developing institutions retain decision power across all stages.

No VAA specifies a mechanism to allow the flagging of issues related to bias, discrimination or poor performance (R5.7) or offer a definition of fairness (R5.8).

Wahl-O-Mat goes through great efforts to be accessible to the visually impaired German-speaking population, offering a sign language and audio function as well as a version in Easy German (Bundeszentrale für politische Bildung, 2023). StemWijzer is explicitly designed for those who are visually impaired (Slik, 2023) and under consideration of Simple Dutch. No other VAA mentions accessibility or universal design (R5.9, R5.10).

Smartvote and StemWijzer provide translations into English for some questionnaire versions only, not extending to documentation. Other VAAs support only main national languages (R5.11).

### R6: societal and environmental well-being

VAA scores on societal and environmental well-being are similar. Issues that may result in harm to essential areas such as environment, societal welfare, rule of law and democracy are inherently political and covered as content of the VAA questionnaires, but are not discussed further (R6.1). No VAA publicly discusses potential societal impacts (R6.2) nor efforts of mitigating possible harm (R6.3) or impact on election outcomes (R6.4). Only HS Vaalikone mentions a possible effect on political representation in a newspaper article on a party’s potentially strategic answering pattern (Junkkari, 2023) (R6.5).

Smartvote and Kieskompas What2Vote have strong ties with academia and collaborate closely with research (see e.g. Ladner (2016); Kieskompas BV (2024d)). Wahl-O-Mat collaborates with the Heinrich Hesse Universität Düsseldorf and with the Wahl-O-Mat Forschung Düsseldorf, though the extent of this collaboration is unclear (Bundeszentrale für politische Bildung, 2023). Similarly, while SVT Nyheters valkompass, Aftonbladets valkompass and StemWijzer are designed in collaboration with academia, their scientific contributions are unclear. HS Vaalikone does not disclose scientific involvement.

### R7: accountability

No VAA clearly defines mechanisms to identify relevant interests and values that may be implicated, though the participatory design process of the Wahl-O-Mat can be considered to serve this purpose (R7.1). HS Vaalikone mentions trade-offs relating to the switch to value-based party positioning (Nurmela & Hulst, 2023).

SVT Nyheters valkompass and Aftonbladets valkompass provide a dedicated point of contact to report errors which may be extended to potential vulnerabilities, risks or biases (R7.2). Smartvote hosts a contact form for general communication. The other VAAs provide institutional email addresses.

The process of candidates redressing their political placement depends on the method used in the first place (R7.4). While academic literature refers to an authorization of political placement within Kieskompas by parties (Krouwel & van Elfrinkhof, 2014), the FAQ makes no mention thereof. Similarly, party placement within Aftonbladets valkompass is discussed bilaterally with parties. The other VAAs use self-placement, verification of which is only mentioned by StemWijzer (Bamberg et al., 2021). HS Vaalikone allows candidates to change their answers at any time. The other VAAs do not provide this option.

Only Kieskompas undergoes an annual privacy audit by an external agency (BV, 2024b) (R7.5). No other VAA provides information on external guidance, on procedures for the event of attacks (R7.3), on risk training (R7.6) or on the overseeing of societal effects (R7.7).

## Discussion

None of the VAAs under investigation scored highly on the adapted EGTAI assessment list. For several requirements, many sub-requirements are not fulfilled by any VAA in this study. In particular, scores on societal and environmental well-being (R6) or accountability (R7) are low without significant differences between VAAs.

StemWijzer and Wahl-O-Mat, characterized by low algorithmic complexity and results presented as a ranked list, score comparatively highly on the requirements of diversity, non-discrimination and fairness (R5). Inclusion is a clear focal point of Wahl-O-Mat’s participatory design framework. However, not even Wahl-O-Mat provides information on stakeholder diversity or the notion of fairness. Both VAAs also score highly on technical robustness and safety (R2) despite using party or candidate answers for question selection (StemWijzer, 2023; Bundeszentrale für politische Bildung, 2023) to ensure distinctive answers. This could be abused to tamper with the probability of which questions are included in the final questionnaire. The focus on simplicity and explicability is not directly reflected in the transparency scores, where only Wahl-O-Mat achieves relatively high marks.

Kieskompas What2Vote performs weakly across requirements. Despite detailed description in academic literature, little information is provided to the lay user such that rigorous effort in standardizing questionnaire development and political placement are not reflected in the EGTAI scores. Aftonbladets valkompass, the second VAA relying on expert placement, puts a greater focus on user-centered documentation and on the separation of decision power across different stakeholders.

Smartvote and HS Vaalikone use complex methods and offer several result interpretations. Their performance varies strongly: Smartvote scores relatively highly on transparency (R4), describing calculation, processing steps, and their interpretation in an easily accessible way. At the same time, Smartvote attains no points on human agency and oversight (R1), referring to the VAA output as politically neutral and purely mathematical. HS Vaalikone, the second representative of the Smartvote family, suffers from a lack of documentation: information is dispersed across newspaper articles with many design aspects not available or hidden in code.

SVT Nyheters valkompass, which falls between the StemWijzer and Smartvote families, shows average performances across requirements.

Scores on privacy and data governance (R3) show the strongest variation. This may be explained by the goals of the developing institution. The top performer Wahl-O-Mat is developed by a federal agency. The public funding may allow it to strongly prioritize privacy, storing the minimal possible amount of information. Kieskompas What2Vote, Aftonbladets valkompass and HS Vaalikone, on the other hand, are published by private enterprises who must generate revenue. Smartvote, StemWijzer and SVT Nyheters valkompass fall well in the middle of these scores, being developed by institutions who are publicly subsidized but still must act economically.

The overall performance of VAAs on the EGTAI is notably weak. This may stem from the evaluation based only on publicly available information. It is likely that several aspects were discussed internally in VAA development but were not documented comprehensively. There are legitimate reasons not to make certain topics available: security-related information may be abused by attackers, the extensive discussion of risks and limitations may deter users from engaging with the VAA at all due to the lack of trust, and too much information may lead to excessive cognitive workload. At the same time, VAAs have a core interest in increasing trust and trustworthiness, requiring a higher degree of openness.

### Recommendations

We identify four main areas where there is room and need for improvement: (i) transparency of and clear communication regarding VAA subjectivity, (ii) diversity of stakeholder participation, (iii) seeing through sizable efforts made in ethical development with user-centric documentation, and (iv) a disclosure of the values and assumptions underlying the VAA.

#### Subjectivity

Firstly, the user needs to be aware of the subjectivity inherent to VAAs to be able to retain informational independence. No VAA in our analysis explicitly discusses the open question of how to evaluate performance and fairness, although Aftonbladets valkompass, SVT Nyheters valkompass and Wahl-O-Mat discuss factors relevant in making an electoral decision beyond issue agreement. Without transparency on VAA subjectivity, a user may lose trust in VAAs after comparing two alternative applications and receiving diverging results. The subjectivity inherent in an electoral recommendation as presented by VAAs must be acknowledged explicitly and prominently.

Secondly, in light of the high impact of design decisions on the VAA result, we recommend to provide the user with several result presentations based on alternative algorithmic procedures. Three VAAs (Smartvote, Aftonbladets valkompass and HS Vaalikone) present at least two alternative representations of results using different spatial models (Politools, 2023; Aftonbladet et al., 2022; Helsingin Sanomat, 2023). Other alternative VAA designs could focus on some of the other factors we use to come to an electoral decision, such as perceived political competence or honesty. Through offering several alternative sources of information, VAAs could distance themselves from the self-portrayal of advisors and take up the more humble role of informers.

#### Diversity of stakeholders

The EGTAI call for the involvement of stakeholders in the development of a system which may affect them directly or indirectly. While some VAAs include participatory elements, little attention is given to diversity beyond accessibility in any VAA under investigation, be it economic, social, cultural, religious, geographic, or in terms of sexuality and gender expression. As such minorities are likely to have different foci and needs in politics, their participation in the development process is likely to have a large impact on thematic selection and application design.

#### User-centric documentation

Several VAAs make laudable and innovative efforts towards ethical design which are not reflected in the EGTAI scores due to the lack of user-centric documentation. Wahl-O-Mat uses a co-design approach where questionnaire development is done by an editorial board consisting of several stakeholders. However, the content of the workshops conducted by the editorial board remains elusive to the user. Similarly, the questionnaire design of Aftonbladets valkompass is entirely in the hands of academic researchers, limiting the power of the publishing media house, but is not described further. The open-sourced algorithms of HS Vaalikone and Aftonbladets valkompass are transparent mostly to the limited user groups able to interpret the code. These VAAs also lean towards more complex methods of calculating matches, merging several types of questionnaire items which are treated differently. Kieskompas What2Vote relies on semi-automated methods for questionnaire development to add objectivity.

While we applaud these important steps, they are not sufficient to ensure public trust and democratic legitimacy. We urge developers to maintain a user-centric view in the choice and description of design processes ensuring transparency and explicability, even when participatory, when conducted by academic researchers, and when code is available publicly.

#### Disclosure of values and assumptions

Lastly, all VAAs we examined share a common set of assumptions and values that correspond to social choice theory (Fossen & Anderson, 2014). This is a limited lens through which to look at democracy, neglecting the active role of interest groups and organizations (Dahl, 2008), the formative and synergetic role of deliberation (Habermas, 1989), and the emotive role of tensions and conflict (Mouffe, 2005). Applications designed for the values underlying alternative paradigms of democracy would likely manifest in very different designs. An alternative core assumption could be that the political landscape has become too fractured and too complex to understand without interactive tools. Such an interpretation calls for more radical solutions to the problem of tackling political complexity. We call for an open and explicit disclosure of the assumptions underlying the VAA. There is a need for a participatory and country-level discourse on values we wish for in applications for political guidance rather than accepting the values integrated into the tools at our disposal now. As argued by Selbst et al. (2019), “the foundations of liberal society depend on the idea that some concepts will be fundamentally contestable and will shift over time, that communities should be allowed to collectively define norms and laws. To set them in stone - or in code - is to pick sides, and to do so without transparent process violates democratic ideals.”

### Limitations

Our study comes with several limitations. Firstly, while the cases were selected to broadly cover most common design variations within Europe, they may not be representative of the European VAA landscape. Findings may not generalize to other applications or across political systems. Secondly, the VAAs under investigation are not typical AI systems and may not be covered comprehensively by the EGTAI. The EGTAI must be interpreted in the context of VAAs, which may introduce bias and subjectivity. The scoring of the VAAs carries a similarly subjective element. Thirdly, AI ethics is a rapidly evolving field, and new guidelines or revisions could affect the relevance and timeliness of this analysis. Finally, our analysis is restricted to information that is publicly available and easily retrievable. This methodological choice does not acknowledge the research efforts made by VAA developers who often have discussed their methodology extensively in academic literature.

## Conclusion

While most VAAs diligently incorporate quality controls and transparently communicate main elements within design process and algorithm, compliance scores with the EGTAI are poor.

Particularly in sight of the upcoming AI Act, our results stress the dire need for stricter regulation and policy support for ethical and trustworthy development.

We see several important questions and goals for future research: (i) a creative exploration of political guidance systems designed according to different models of democracy, (ii) an expansion of participatory methods towards value-based co-development, (iii) the definition of methods to evaluate political recommendations beyond electoral intent pre-use, and (iv) an exploration of the reasons and complexity of the current political sphere.

While these challenges are formidable, their urgency and exigence is indisputable. Future developments are sure to hold key implications for politics and the electorate.

## Supplementary Information

Below is the link to the electronic supplementary material.

Supplementary file 1 (pdf 182 KB)

## Data Availability

The code accompanying this paper will be made available on Github (https://github.com/ethz-coss/vaa-egtai-compliance) upon acceptance of this paper.
