# Supplementary material for: Trustworthiness of voting advice applications in Europe
Source: Ethics Inf Technol. 2024 Aug 12;26(3):55. doi: 10.1007/s10676-024-09790-6 (PMC11415416; doi:10.1007/s10676-024-09790-6)
Supplement: Supplementary file 1 — (pdf 182 KB) [file 10676_2024_9790_MOESM1_ESM.pdf]

Supplementary Information for: Trustworthiness of Voting Advice Applications in Europe

Elisabeth Stockinger[1*], Jonne Maas[2], Christofer Talvitie[3], Virginia Dignum[4]

[1*]ORCID: 0000-0003-1139-591X, Computational Social Science, ETH Zurich, Zurich, Switzerland.
[2]ORCID: 0000-0001-9473-6307, Values, Technology and Innovation, Delft University of Technology, Delft, Netherlands.
[3]School of Communication Research, University of Amsterdam, Amsterdam, Netherlands.
[4]ORCID: 0000-0001-7409-5813, Computing Science, Umeå University, Umeå, Sweden.

*Corresponding author(s). E-mail(s): estockinger@ethz.ch;
Contributing authors: j.j.c.maas@tudelft.nl; c.p.i.talvitie@uva.nl; virginia@cs.umu.se;

Table A1: Details on case selection and document corpus

(a) Elections and document corpus considered in analysis. Open-source code repositories are consulted when available. If application development is outsourced, we additionally consult the information published by the corresponding agency. The listed primary domains include all subsites. The link hosting the respective applications is given in bold.

	**Elections (2023)**	**Document Corpus**
StemWijzer	Dutch provincial parliament	**stemwijzer.nl**<br>prodemos.nl<br>slik.nl/portfolio/stemwijzer/
Kieskompas What2Vote	Greek parliament	**what2vote.gr**<br>kieskompas.nl<br>home.kieskompas.nl
Smartvote	Government council Basel-Landschaft, Switzerland	**smartvote.ch**<br>politools.net
Wahl-O-Mat	City parliament Bremen, Germany	**wahl-o-mat.de**<br>www.bpb.de<br>3pc.de/projekte/millionenfach-im-einsatz-unser-wahl-o-mat-design
SVT Nyheters valkompass	Swedish Riksdag	**valkompass.svt.se**<br>svt.se<br>github.com/svt/election-compass-match
Aftonbladets valkompass	Swedish Riksdag	**aftonbladet.se/valkompassen**<br>aftonbladet.se
HS Vaalikone	Finnish parliament	**vaalikone.fi**<br>hs.fi<br>github.com/Sanoma/sanoma-election-machine-algorithms

(b) Developing institutions of selected VAAs

	**Developing Institution**
StemWijzer	*ProDemos:* a politically neutral organisation with the mission of furthering civic education and supporting democracy subsidized by Dutch governmental bodies. The internet agency *Slik* contributed to web design and development.
Kieskompas What2Vote	*Kieskompas:* Dutch private research agency with a focus on political and societal opinion mapping using quantitative methods founded at the University of Amsterdam which has launched VAAs throughout the world (Kieskompas BV, 2024).
Smartvote	*Politools*: a politically neutral non-profit scientific network involved in civic education as well as research projects in the field of e-democracy and VAAs.
Wahl-O-Mat	*Bundeszentrale für politische Bildung (bpb):* German Federal Agency for Civic Education. The digital agency *3pc* contributed to web design and development.
SVT Nyheters valkompass	*Sveriges Television AB (SVT):* Swedish national public broadcaster funded by a public service tax on personal income.
Aftonbladets valkompass	*Aftonbladet:* newspaper publisher owned by a media house (Aftonbladet, 2016).
HS Vaalikone	*Helsingin Sanomat:* a newspaper publisher owned by the media house Sanoma.

Table A2: Common design characteristics and variations as described by Garzia and Marschall (2019).

**Characteristic**	**Common variations**
Content type	Political issues, values
Statement selection	usually conducted by experts such as journalists or political scientists, but may include party actors or voters
Number of statements	usually around 25 to 30, but can be twice that
Inclusion criteria	the parties or candidates to be represented in the VAA may be restricted, e.g. to those parties with a seat in parliament
Answer categories	typically a scale from 3 (agree, disagree, neutral) to 5 options (e.g. a Likert-scale from strong agreement to strong disagreement), sometimes without a neutral position

Table A3: Design characteristics of the selected VAAs. The ✓ in column *Skip* indicates that questions can be skipped. Column *Weights* lists factors that may be applied to questionnaire items, assuming a default weight of 1. Weights > 1 assign higher priority to a question while weights < 1 represent low priority.

	**Statement selection**	**Number of statements**	**Inclusion criteria**	**Answer categories**	**Skip**	**Weights**	**Distance algorithm**
StemWijzer	Developing institution	30	All	3-point scale	✓	2	Exact match
Kieskompas What2Vote	Developing institution	30	Parliamentary party or running for at least one seat	5-point Likert scale	✓		Not applicable
Smartvote	Developing institution	30/59[a]	All	4-point scale	✓	$\frac{1}{2}$, 2	Euclidean
Wahl-O-Mat	Developing institution, experts, voters	38	All	3-point scale	✓	2	Manhattan
SVT Nyheters valkompass	Developing institution, scientists	50	Parliament party	4-point scale, 5-point range, multiple-choice	✓	4[b]	Ratio of maximum score
Aftonbladets valkompass	Scientists	30	Parliament party	5-point Likert scale, hidden neutral option	✓		Manhattan
HS Vaalikone	Developing institution	30	All	5-point Likert scale	✓		Manhattan (issues), Euclidean (values)

[a] Available in a "rapid" and "deluxe" version.
[b] Limited to at most 5 items.

Table A4: Compliance of each VAA with each sub-requirement.

Sub-requirement	Scale	StemWijzer	Kieskompas What2Vote	Smartvote	Wahl-O-Mat	Aftonbladets valkompass	SVT Nyheters valkompass	HS Vaalikone
R1.1	(0) Not fulfilled/no information, (1) fulfilled	0	0	0	0	0	0	0
R1.2	(0) Not fulfilled/no information, (1) implicit attention, (2) explicit identification and documentation	0	0	0	1	0	0	1
R1.3	(0) Not fulfilled/no information, (1) fulfilled	0	0	0	0	0	0	0
R1.4	(0) Not fulfilled/no information, (1) some measures (such as rejecting the claim to be an electoral recommendation), (2) significant measures (such as suggesting alternative factors to consider in an electoral decision)	1	1	0	2	2	2	0
R2.1	(0) Self-placement only, (1) internal validation, (2) expert validation	1	2	0	0	2	0	1
R2.2	One point is given for each: (i) expert involvement, and (ii) user involvement in validation	2	1	2	2	2	2	1
R2.3	One point is given if a measure was implemented	1	0	0	1	0	0	0
R2.4	(0) Not fulfilled/no information, (1) mentioned, (2) thorough documentation	0	0	0	0	0	0	0
R2.5	One point is given for the support of (i) exclusively TLS 1.2 and up, (ii) HTTP Strict Transport Security, (iii) only cipher suits without known vulnerabilities, and (iv) DNS Certification Authority Authorization	4	4	0	2	2	0	3
R2.6	One point is given for (i) well-configured security headers, (ii) secure cookie settings, and (iv) no version-based vulnerabilities	1	1	2	2	1	2	2
R2.7	(0) Not fulfilled/no information, (1) anecdotal information (2) clear procedure	0	0	0	0	0	0	1
R2.8	(0) Not fulfilled/no information, (1) thorough testing across scenarios and user groups	0	0	0	0	0	0	0
R3.1	(0) Collects data on the user-level by default/no information, (1) collects only quantitative usage data, (2) collects only technical data	1	1	2	2	1	1	1
R3.2	(0) Funded through advertisement, (1) by providing additional services, (2) publicly funded	2	0	1	2	0	2	0
R3.3	(0) Not fulfilled, (1) aggregation, (2) no personal data is collected	1	1	1	2	1	1	1
R4.1	One point is given for every representation	1	1	3	1	2	1	2
R4.2	(0) Not fulfilled, (1) answers are available, (2) question-wise comparisons are available	2	2	1	2	2	2	2
R4.3	(0) Not fulfilled, (1) fulfilled	1	1	1	1	1	1	1
R4.4	(0) Not fulfilled, (1) some design decisions are documented, (2) extensive documentation including reasons and actors	1	0	2	1	1	2	0
R4.5	(0) Not fulfilled (1) open-source algorithm, (2) clear communication to end-user and non-technical experts	2	0	2	2	2	1	1
R4.6	(0) Not mentioned, (1) implicit communiation, (2) explicit communication	1	1	2	1	2	2	1
R4.7	(0) Not fulfilled, (1) implicit communication, (2) explicit communication	1	1	0	0	0	0	0
R4.8	(0) Not fulfilled, (1) implicit communciation, (2) explicit communication	0	0	0	2	0	0	1
R4.9	(0) Not fulfilled, (1) implicit communciation, (2) explicit communication	1	0	0	1	2	1	0
R4.10	(0) Not fulfilled, (1) implicit communciation, (2) explicit communication	0	0	0	2	2	2	1
R4.11	(0) Not fulfilled, (1) fulfilled	1	1	1	1	1	1	1
R5.1	(0) Not clear/not communicated, (1) communicated on the FAQ only, (2) clearly communicated on the VAA app or all parties are included	0	1	2	2	1	1	0

Continued on next page

Table A4: Compliance of each VAA with each sub-requirement. (Continued)

Sub-requirement	Scale	StemWijzer	Kieskompas What2Vote	Smartvote	Wahl-O-Mat	Aftonbladets valkompass	SVT Nyheters valkompass	HS Vaalikone
R5.2	One point is given for each (i) public call for questions, and subsequent (ii) user, (ii) expert or (iv) party involvement	4	0	3	2	1	2	1
R5.3	One point is given if any party other than the developing institution holds decision power at any stage	0	0	0	1	1	0	0
R5.4	(0) Not fulfilled/no information, (1) implicitly considered, (2) clear focal point and explicitly discussed	0	0	0	1	0	0	0
R5.5	(0) Not fulfilled/no information, (1) fulfilled	0	0	0	0	0	0	0
R5.6	(0) Not fulfilled/no information, (1) non-binding or internal measures, (2) binding expert involvement	1	2	1	1	2	1	1
R5.7	(0) Not fulfilled, (1) fulfilled	0	0	0	0	0	0	0
R5.8	(0) Not fulfilled, (1) fulfilled	0	0	0	0	0	0	0
R5.9	(0) Not fulfilled/no information, (1) implicitly, (2) clear focal point and explicitly discussed	1	0	0	1	0	0	0
R5.10	(0) Not fulfilled/no information, (1) implicitly, (2) clear focal point and explicitly discussed	2	0	0	2	0	0	0
R5.11	One point is given if the VAA is available any language other than the main national one(s)	1	0	1	0	0	0	0
R6.1	(0) Not fulfilled/no information, (1) by content topic, (2) additional factors	1	1	1	1	1	1	1
R6.2	(0) Not fulfilled/no information, (1) internal assessment, (2) external assessment	0	0	0	0	0	0	0
R6.3	One point is given if an action has been taken	0	0	0	0	0	0	0
R6.4	(0) Not fulfilled/no information, (1) general information, (2) contextual information	0	0	0	0	0	0	0
R6.5	(0) Not fulfilled/no information, (1) some communication, (2) extensive communication	0	0	0	0	0	0	1
R6.6	(0) Not fulfilled/no information, (1) collaboration with reseachers, (2) contribution to publications	1	2	2	1	1	1	0
R7.1	One point is given for (i) the definition of values, and (ii) mechanism, respectively	0	0	0	1	0	0	1
R7.2	(0) Not fulfilled/no information, (1) generic/ institutional contact information, (2) dedicated contact point	1	1	1	1	2	2	1
R7.3	(0) Not fulfilled/no information, (1) clear procedure	0	0	0	0	0	0	0
R7.4	(0) Change is possible at any time without expert approval, (1) change not possible, (2) redress is possible according to a clear procedure	1	1	1	1	1	1	0
R7.5	(0) Not fulfilled/no information, (1) external guidance at some point, (2) external guidance throughout the entire lifecycle	0	1	0	0	0	0	0
R7.6	(0) Not fulfilled/no information, (1) risk training was organised and (2) included legal frameworks	0	0	0	0	0	0	0
R7.7	(0) Not fulfilled/no information, (1) fulfilled	0	0	0	0	0	0	0

Table A5: Compliance of each VAA over the key requirements in the EGTAI as the mean and standard deviation over compliance across the corresponding sub-requirements.

Nr.	StemWijzer		Kieskompas What2Vote		Smartvote		Wahl-O-Mat		Aftonbladets valkompass		SVT Nyheters valkompass		HS Vaalikone	
	μ	σ^2	μ	σ^2	μ	σ^2	μ	σ^2	μ	σ^2	μ	σ^2	μ	σ^2
R1	0.12	0.25	0.12	0.25	0.00	0.00	0.38	0.48	0.25	0.50	0.25	0.50	0.12	0.25
R2	0.50	0.46	0.38	0.44	0.25	0.46	0.44	0.50	0.38	0.44	0.25	0.46	0.41	0.38
R3	0.67	0.29	0.33	0.29	0.67	0.29	1.00	0.00	0.33	0.29	0.67	0.29	0.33	0.29
R4	0.59	0.38	0.41	0.44	0.64	0.55	0.73	0.34	0.77	0.41	0.68	0.40	0.55	0.42
R5	0.36	0.45	0.14	0.32	0.30	0.43	0.45	0.42	0.25	0.40	0.14	0.23	0.07	0.16
R6	0.17	0.26	0.25	0.42	0.25	0.42	0.17	0.26	0.17	0.26	0.17	0.26	0.17	0.26
R7	0.14	0.24	0.21	0.27	0.14	0.24	0.21	0.27	0.21	0.39	0.21	0.39	0.14	0.24

Table A6: VAA support for protocols, cipher suites and standards (R2.5) as well as website configuration (R2.6) related to security. Support for vulnerable technologies is denoted with ×, implementation of security-promoting technologies with ✓.

		Smartvote	Wahl-O-Mat	SVT Nyheters valkompass	Aftonbladets valkompass	HS Vaalikone	StemWijzer	Kieskompas What2Vote
HTTP Strict Transport Security (HSTS)[a]					✓	✓	✓	✓
DNS Certification Authority Authorization (DNS CAA)[b]							✓	
Set-Cookie with *HttpOnly* flag[c]		✓	✓	✓		✓	✓	
Set-Cookie restrictive *Domain* flag[c]		✓	✓	✓		✓	✓	✓
Transport Layer Security (TLS) $\leq$ 1.1[d]		×		×				
Version-based software vulnerability							×[e]	
Cipher suites	RSA[f]	×			×			
	CBC[g]	×	×	×	×			
	Triple-DES[h]	×						
	SHA[i]	×		×				

[a] HSTS allows web servers to restrict interaction to HTTPS (Hodges et al, 2012).
[b] DNS CAA reduces the risk of unintended certificate misuse (Hallam-Baker et al, 2019).
[c] The *HttpOnly* and *Domain* flags are optional attributes in the $Set-cookie$ response header. *HttpOnly* helps mitigate the risk of client side script accessing the protected cookie. If *Domain* is set too permissively, attacks may be launched on session IDs between different hosts and web applications belonging to the same domain (Open Worldwide Application Security Project (OWASP), 2024).
[d] TLS versions 1.0 and 1.1 were deprecated due to lack of current recommended cryptography algorithms and mechanisms (Moriarty and Farrell, 2021).
[e] At time of analysis the website used Bootstrap 3.3.7 which is vulnerable to cross-site scripting (snyk, 2024).
[f] This column denotes use of Rivest Shamir Adleman algorithm (RSA) for key exchange. As RSA does not ensure Perfect Forward Secrecy, key exposure compromises the secrecy of the complete communication stream (Krawczyk, 2005).
[g] Cipher Block Chaining (CBC) encryption is vulnerable to timing attacks (Al Fardan and Paterson, 2013).
[h] Triple-DES encryption uses the relatively small block size of 64 (Bhargavan and Leurent, 2016).
[i] Secure Hash Algorithm 1 (SHA) is vulnerable to collision attacks (Stevens et al, 2017).

References

Aftonbladet (2016) About aftonbladet - contact and editorial policy. https://web.archive.org/web/20230629130342/https://www.aftonbladet.se/omaftonbladet/a/LO1Q4/om-aftonbladet, accessed: 2023-06-29

Al Fardan NJ, Paterson KG (2013) Lucky thirteen: Breaking the TLS and DTLS record protocols. In: 2013 IEEE Symposium on Security and Privacy, pp 526–540, https://doi.org/10.1109/SP.2013.42

Bhargavan K, Leurent G (2016) On the practical (in-)security of 64-bit block ciphers: Collision attacks on HTTP over TLS and OpenVPN. In: Proceedings of the 2016 ACM SIGSAC Conference on Computer and Communications Security. Association for Computing Machinery, New York, NY, USA, CCS '16, p 456–467, https://doi.org/10.1145/2976749.2978423

Garzia D, Marschall S (2019) Voting advice applications. https://doi.org/10.1093/acrefore/9780190228637.013.620

Hallam-Baker P, Stradling R, Hoffman-Andrews J (2019) DNS Certification Authority Authorization (CAA) Resource Record. RFC 8659, https://doi.org/10.17487/RFC8659

Hodges J, Jackson C, Barth A (2012) HTTP Strict Transport Security (HSTS). RFC 6797, https://doi.org/10.17487/RFC6797

Kieskompas BV (2024) Kieskompas worldwide. https://www.kieskompas.nl/nl/kieskompas/kieskompas-worldwide/, accessed: 2024-05-28

Krawczyk H (2005) Perfect Forward Secrecy, Springer US, Boston, MA, pp 457–458. https://doi.org/10.1007/0-387-23483-7_298

Moriarty K, Farrell S (2021) Deprecating TLS 1.0 and TLS 1.1. RFC 8996, https://doi.org/10.17487/RFC8996

Open Worldwide Application Security Project (OWASP) (2024) Session management. https://cheatsheetseries.owasp.org/cheatsheets/Session_Management_Cheat_Sheet.html

snyk (2024) bootstrap@3.3.7 vulnerabilities. https://security.snyk.io/package/npm/bootstrap/3.3.7

Stevens M, Bursztein E, Karpman P, et al (2017) The First Collision for Full SHA-1. In: Katz J, Shacham H (eds) Advances in Cryptology – CRYPTO 2017. Springer International Publishing, pp 570–596, https://doi.org/10.1007/978-3-319-63688-7_19
